# Comprehensive Analysis of CDCAs Methylation and Immune Infiltrates in Hepatocellular Carcinoma

**DOI:** 10.3389/fonc.2020.566183

**Published:** 2021-02-16

**Authors:** Yongkang Wang, Yinfeng Yang, Honglei Gao, Ting Ouyang, Luyao Zhang, Jili Hu, Sheng Hu, Hongxing Kan

**Affiliations:** ^1^ School of Medical Informatics Engineering, Anhui University of Chinese Medicine, Hefei, China; ^2^ Anhui Computer Application Research Institute of Chinese Medicine, China Academy of Chinese Medical Sciences, Hefei, China

**Keywords:** hepatocellular carcinoma, CDCA, DNA methylation, immune infiltrates, predictive biomarker

## Abstract

**Background:**

As essential components of cycle growth, the cell division cycle-associated family genes (CDCAs) have crucial roles in tumor development and progression, especially in hepatocellular carcinoma (HCC). However, due to the tumor heterogeneity of HCC, little is known about the methylation variability of CDCAs in mediating phenotypic changes (e.g., immune infiltrates) in HCC. Presently, we aim to comprehensively explore the expression and prognosis of CDCAs methylation with regard to immune infiltrates of HCC.

**Methods:**

We first identified the correlating differentially expressed genes (co-DEGs) among 19 different types of cancer cohorts (a total of 7,783 patients) and then constructed the weighted gene co-expressed and co-methylated networks. Applying the clustering analysis, significant modules of DEGs including CDCAs were selected and their functional bioinformatics analyses were performed. Besides, using DiseaseMeth and TIMER, the correlation between the methylation levels of CDCAs and tumor immune infiltrates was also analyzed. In final, to assess the influence of CDCAs methylation on clinical prognosis, Kaplan-Meier and Cox regression analysis were carried out.

**Result:**

A total of 473 co-DEGs are successfully identified, while seven genes of CDCAs (CDCA1–3 and CDCA5–8) have significant over-expression in HCC. Co-expressed and co-methylated networks reveal the strong positive correlations in mRNA expression and methylation levels of CDCAs. Besides, the biological enrichment analysis of CDCAs demonstrates that they are significantly related to the immune function regulation of infiltrating immune cells in HCC. Also, the methylation analysis of CDCAs depicts the strong association with the tumor immunogenicity, i.e., low-methylation of CDCA1, CDCA2, and CDCA8 dramatically reduced the immune infiltrate levels of T cells and cytotoxic lymphocytes. Additionally, CDCA1–6 and CDCA8 with low-methylation levels significantly deteriorate the overall survival of patients in HCC.

**Conclusions:**

The co-expressed and co-methylated gene networks of CDCAs show a powerful association with immune function regulation. And the methylation levels of CDCAs suggesting the prognostic value and infiltrating immune differences could be a novel and predictive biomarker for the response of immunotherapy.

## Introduction

In accordance with the global cancer statistics released in 2018, hepatocellular carcinoma (HCC) has become the sixth most common cancer worldwide and the second leading cause of cancer-related death in men (1). Although several established treatments, including surgical resection, chemotherapy, and radiofrequency ablation, have been applied to the patient with HCC, the long-term survival of patients remains poor (2). Therefore, research into novel and effective treatment and prognostic signatures will need to be further performed.

Currently, the evolution and progression of tumors are considered to be related to the abnormal expression of various cancer-related genes, which involves different aspects of the life process in cells (e.g., cell cycle controlling, cell growth, cell apoptosis). Among them, the cell division cycle-associated family genes (CDCAs), consisting of eight members (CDCA1–8), are essential components of cell cycle growth, and they have been found malfunctions in various cancers, including HCC (3–6). For instance, CDCA1, also known as NUF2 (NUF2 component of NDC80 kinetochore complex), could inhibit tumor growth and induce apoptosis in HCC through silencing itself (7). And CDCA2 promoted the proliferation of tumor cells *via* activating the AKT/CCND1 pathway (8). Through enhancing cell proliferation with the prevention of G1 phase arrest, the upregulation of CDCA3 promoted cancer progression (9). With respect to CDCA6, also known as CBX2 (chromobox 2), it could become the regulator of the proliferation and apoptosis by the phosphorylation of YAP (yes1 associated transcriptional regulator) in HCC (10). Besides, CDCA8, as the key mediator of estrogen-stimulated cell proliferation in cancer cells, could inhibit cancer cell survival and growth by cell cycle G1 phase arrest (11).

Additionally, cancer immunotherapy has made tremendous success in cancer treatment, and a more detailed understanding of the immune infiltrate may be beneficial in developing the rationale for immunotherapy (12, 13). Increasing researches have indicated the relation between cancer immunotherapy and DNA methylation (14–16). For example, Gallagher et al. have reported that DNA methylation could contribute to cancer immunotherapy *via* modulating immune cell differentiation and function (15). Notably, several genes of CDCAs have also been involved in the immune infiltrates. Taking CDCA4 as an example, it is the E2F transcription factor, which plays an important role in immunity regulation (17, 18). Also, the expression of CDCA6 in immune cells (e.g. macrophages and T cells) could suggest the intensity of innate immune response (19). Moreover, CDCA7, associated with DNA methylation and cellular functions, has a regulation in the apoptosis of T cells (20, 21). However, despite the closed relation of the CDCAs in the occurrence and development of HCC, the association between the methylation of these genes and immune infiltrates in HCC has not been systematically investigated.

**Figure 1:**
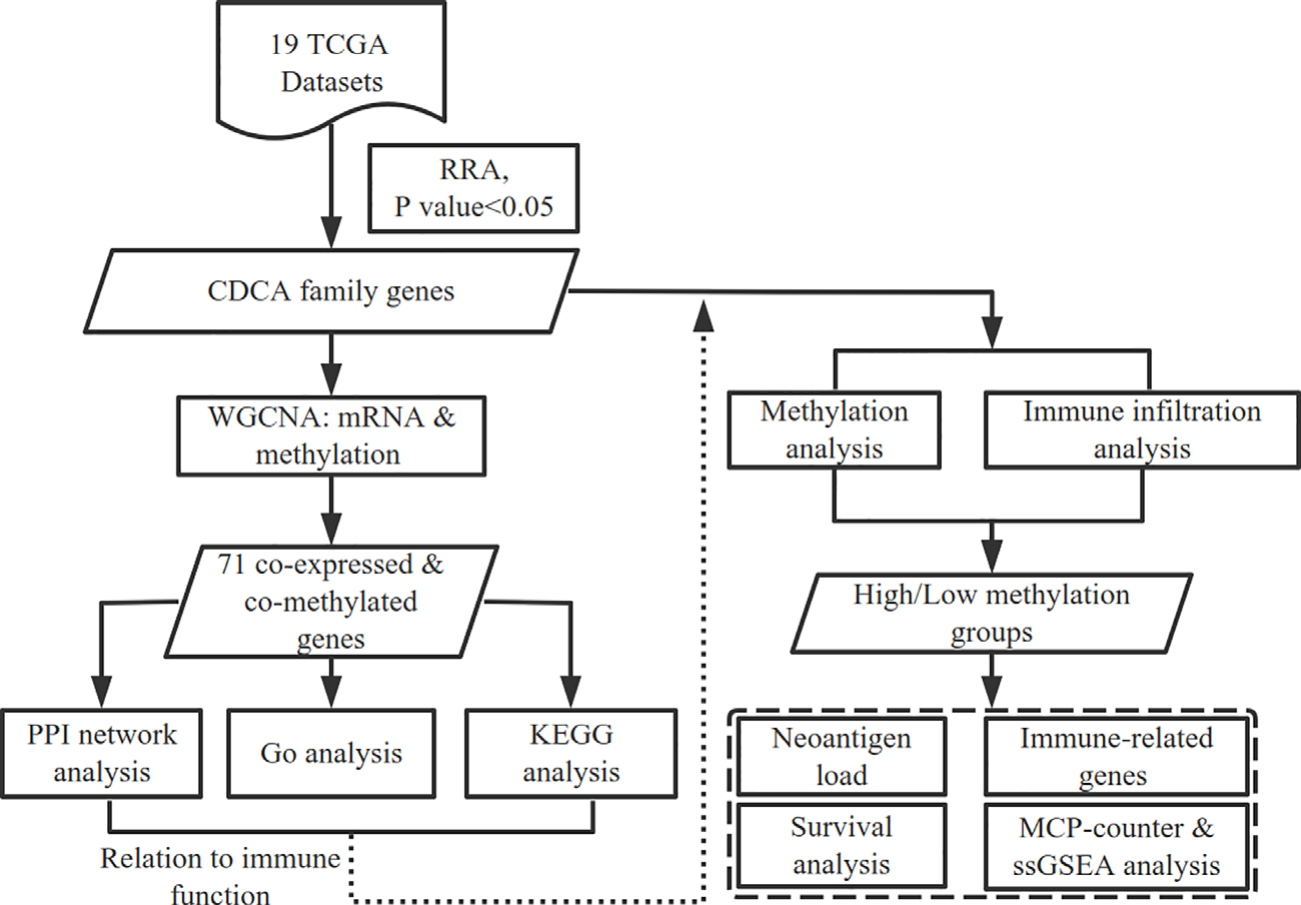
Workflow of this study. The high and low methylation groups are classified by the median methylation levels of CDCAs.

In this study, we obtained plenty of cancer datasets from TCGA and found significantly up-regulated CDCAs in HCC. Through constructing the co-expressed and co-methylated networks, we explored the biological enrichment of CDCAs and found that they are significantly related to immune function. Further, the immune infiltration and prognostic value between the high and low methylation groups of CDCAs were also compared. All the results demonstrate that the methylation levels of CDCAs could be served as predictive biomarkers for determining prognosis and immune infiltration in HCC. The flow diagram of this study was illustrated in **Figure 1**.

## Materials and Methods

### Differentially Expressed Genes (DEGs) Analysis

**Figure 2:**
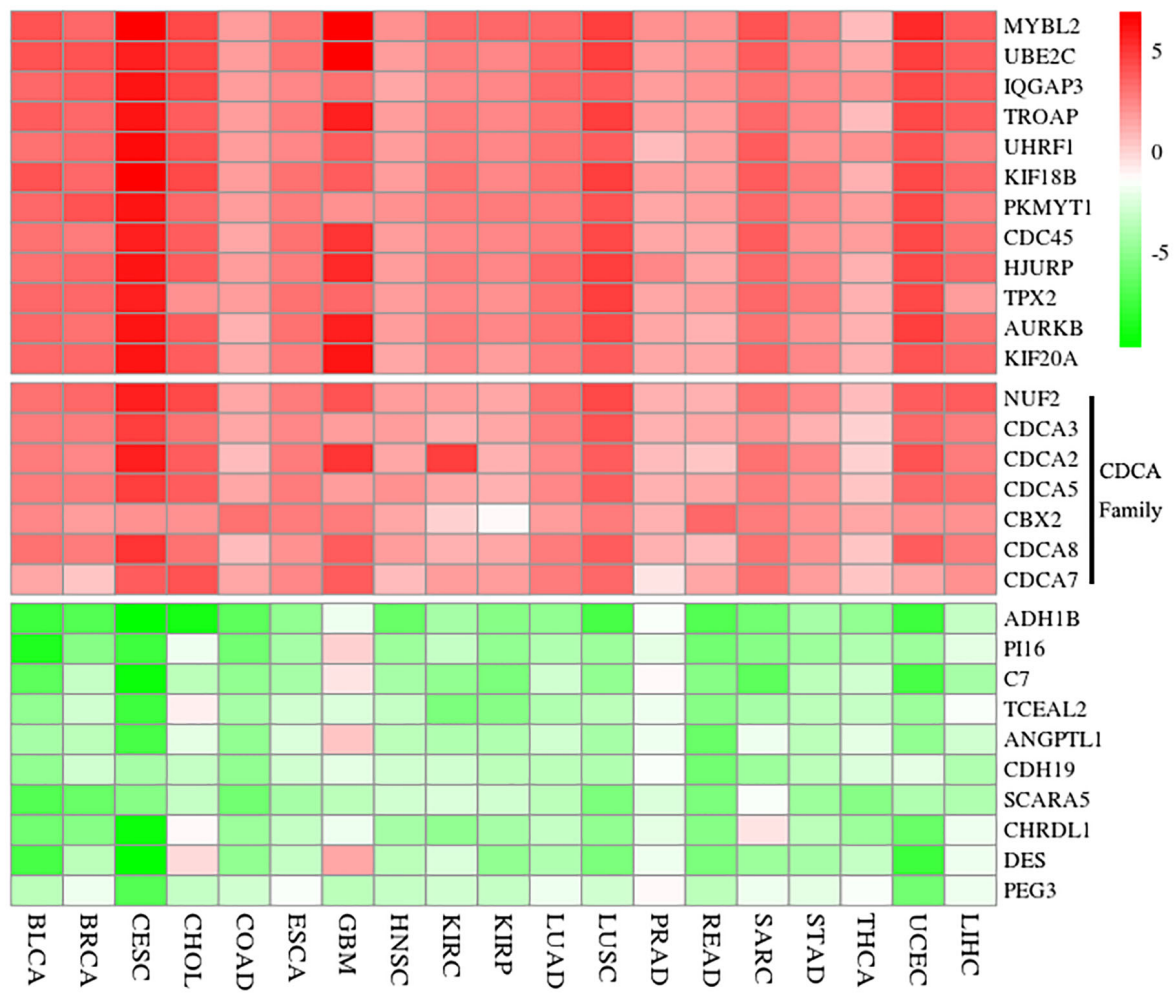
Result of significant DEGs *via* the RRA analysis. Red shows up-regulation and green reveals down-regulation. The value is the logFC from the DEG analysis of each dataset. Additionally, seven genes of CDCAs (NUF2, CDCA2, CDCA3, CDCA5, CBX2, CDCA7, and CDCA8) are up-regulated in each dataset. And the full names of cancer types in x-arias are Bladder Urothelial Carcinoma (BLCA), Breast invasive carcinoma (BRCA), Cervical squamous cell carcinoma and endocervical adenocarcinoma (CESC), Cholangiocarcinoma (CHOL), Colon adenocarcinoma (COAD), Esophageal carcinoma (ESCA), Glioblastoma multiforme (GBM), Head and Neck squamous cell carcinoma (HNSC), Kidney renal clear cell carcinoma (KIRC), Kidney renal papillary cell carcinoma (KIRP), Lung adenocarcinoma (LUAD), Lung squamous cell carcinoma (LUSC), Pancreatic adenocarcinoma (PRAD), Rectum adenocarcinoma (READ), Sarcoma (SARC), Stomach adenocarcinoma (STAD), Thyroid carcinoma (THCA), Uterine Corpus Endometrial Carcinoma (UCEC), and Liver hepatocellular carcinoma (LIHC). logFC, logarithmic fold change; RRA, robust rank aggregation.

Publicly available expression data of HCC were collected from The Cancer Genome Atlas (TCGA, https://cancergenome.nih.gov/). After removing the datasets without enough normal examples, a total of 19 different types of cancer datasets (the detailed descriptions of cancers were listed in **Figure 2**) and 7,783 patients were finally obtained. Meanwhile, to identify the DEGs, we compared the tumor and normal samples of each dataset employing the “limma” R package (22), and the thresholds were set to FDR<0.01 and |log2FC|>2, respectively. Using the R package “RobustRankAggreg” (RRA) (23), the DEGs of these datasets were integrated to explore the correlating differentially expressed genes (co-DEGs). Notably, the genes with adjusted P < 0.05 were sorted by their log2FC into the up-regulated and down-regulated gene lists in the RRA analysis. Additionally, since members of the gene family have similar biological functions, we used the Hugo Gene Nomenclature Committee (HGNC) online database (https://www.genenames.org/) to build the gene families of DEGs and validate the results through literature checking.

### Weighted Gene Co-Expressed and Co-Methylated Networks Construction

The gene expression profile matrix file of patients with HCC, including 374 tumor samples, was publicly obtainable from TCGA. The co-expressed and co-methylated genes were analyzed by the “WGCNA” R package (24). Through setting the parameters of the network analysis (e.g., soft-thresholding = 12, scale-free R^2 = 0.90, and cut-height = 0.25), all the genes were divided into different gene modules according to the mRNA expression matrix. Meanwhile, using the cutoff of |log2FC|>2 and FDR<0.01 for the DEGs analysis in the liver hepatocellular carcinoma (LIHC) dataset, we collected the significant expression genes from the obtained gene modules.

Additionally, the methylation microarray data containing 380 tumor samples were collected from the TCGA-LIHC dataset. After removing the invalid and low-quality expression value, we corrected the deviation of methylation expression *via* the R package ChAMP v2.14.0. With the same parameters of the co-expressed network analysis, the methylation genes were divided into different gene modules. Employing the threshold values of |logFC|>0.2 and p < 0.05, we explored the differential methylation probes (DMP) to choose the significant methylation genes *via* the R package minfi v1.30.0. All the significant genes overlapping in the key expression module and methylation module (25) were used to perform further analysis.

### Protein−Protein Interactions (PPI) Network Construction and Functional Enrichment Analysis

We established the functional protein interaction network for 71 co-expressed and co-methylated genes by using known and predicted interactions through the STRING database (26). Besides, we calculated the betweenness and degree (27) of all significant genes *via* the R package “igraph” v1.2.6. Furthermore, the Database for Annotation Visualization and Integrated Discovery (DAVID) (28) was performed to explore the biological enrichment of these genes. With setting the statistical significance as 0.05, the Gene Ontology (GO) terms and Kyoto Encyclopedia of Genes and Genomes (KEGG) pathway were visualized by R package “GOplot” (29).

### Methylation Analysis

To investigate the methylation levels of CDCAs in normal and tumor samples of HCC, we used the online tools DiseaseMeth (30, 31). It integrates methylation data from several public sources and literature and provides information on the associations between diseases and genes. Herein, we set the technology experimental platform as 450k (Illumina Infinium HumanMethylation 450 BeadChip) and the significant p-value as 0.05. Moreover, to indicate whether the abnormal methylation has an effect on the expression level of CDCAs, we explored the correlation between their expression and methylation levels and visualized this result *via* the R package corrplot v0.84.

### Immune Cell Infiltration Analysis

To explore the association between the mRNA expressions of CDCAs and immune cell infiltration, we used the website service TIMER (32, 33). The TIMER database validated by pathological estimations can systematically analyze the tumor immune infiltration across different cancer types.

### Analyzing the Relationship Between Immune Infiltrates and CDCAs Methylation

Based on the median methylation levels of each CDCAs, the tumor samples were divided into the high and low methylation groups. To explore the tumor immune infiltration of HCC, we further compared the respective associations between the neoantigen load (33), seven classical immune signatures (34), immune-related gene expressions (35), and the methylation groups of CDCAs. We applied the R package MCP-counter v1.1.0 to assess abundances of tissue-infiltrating immune. And the R package GSVA v1.34.0 was applied to investigate single sample GSEA (ssGSEA) enrichment scores of these immune signatures.

### Survival Analysis

To evaluate the associations between DNA methylation and survival of HCC patients, 370 samples with both the clinical and methylation information from the obtained HCC dataset were subjected to further perform cox regression analysis. Additionally, the Kaplan-Meier method was also applied to explore the differences in the survival time of the methylation groups, with the R packages survival v3.1-8 and survminer v0.4.6.

### Statistical Analysis

The multivariate cox regression analysis was employed to investigate the independent prognostic value of other survival features. Log-rank and cox p-value analysis was performed to explore the statistical significance of observed differences in methylation groups. Additionally, the statistical significance was set as 0.05.

## Result

### mRNA Expression Levels of the CDCAs in HCC

According to the result of RRA analysis, we obtained 159 up-regulation and 314 down-regulation differential genes. Among them, seven genes of CDCAs (34) (CDCA1/NUF2: P = 2.73E-22, CDCA2: P = 1.52E-16, CDCA3: p = 1.50E-20, CDCA5: P = 1.77E-20, CDCA6/CBX2: P = 6.87E-19, CDCA7: P = 5.92E-16, and CDCA8: P = 1.67E-18) were all up-regulated in 19 cancer datasets (**Figure 2**). Especially, CDCAs are significantly up-regulated in HCC: CDCA1 (log2FC = 3.72, adjusted P = 6.70E-46), CDCA2 (log2FC = 2.76, adjusted P = 1.4E-29), CDCA3 (log2FC = 2.92, adjusted P = 1.96E-39), CDCA5 (log2FC = 3.15, adjusted P = 2.603E-45), CDCA6 (log2FC = 2.12, adjusted P = 3.79E-21), CDCA7 (logF2C = 2.29, adjusted P = 3.40E-10), and CDCA8 (log2FC = 2.86, adjusted P = 3.21E-39).

**Figure 3:**
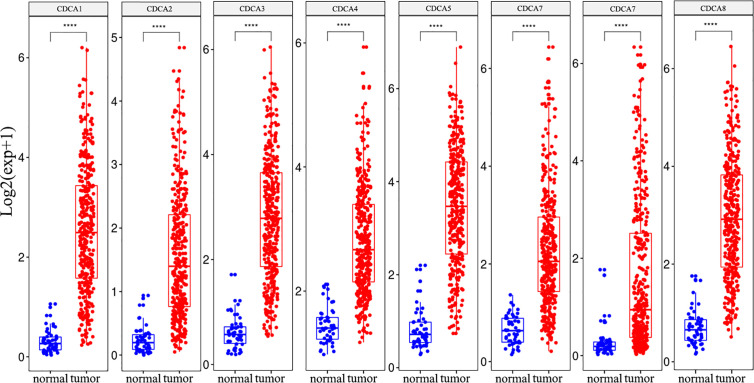
The CDCAs have significant differences between HCC and normal tissue. Meanwhile, eight genes of CDCAs have over-expression in the tumor sample. T-test, ****p < 0.0001.

Subsequently, the mRNA expression levels of CDCAs were explored in the tumor and normal samples from the LIHC dataset. The results indicated that CDCA1–8 were over-expressed in cancer tissues than in normal tissues, and these genes all had significant differences (**Figure 3**).

### Analysis of Co-Expressed and Co-Methylated Networks

**Figure 4:**
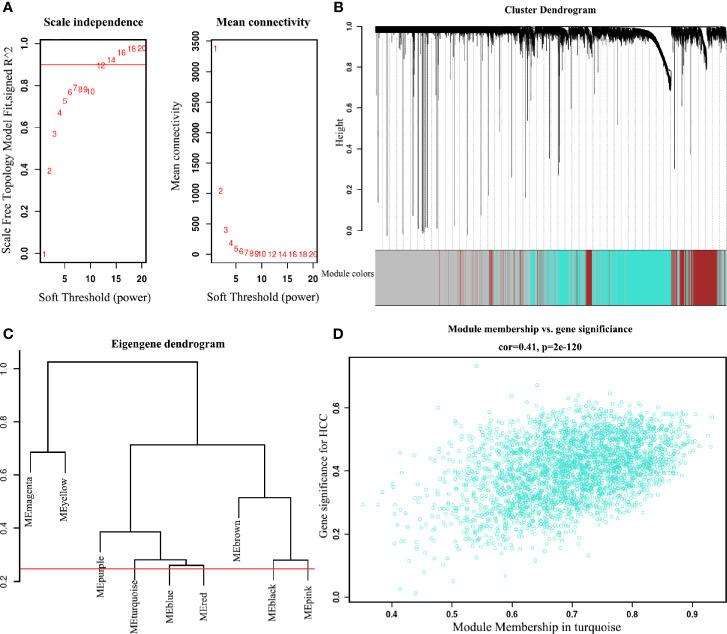
Identify the key module of the LIHC dataset by WGCNA **(A)** Analysis of soft-threshold powers with the scale-free index and the mean connectivity. **(B)** Dendrogram of all filtered genes enrich according to a dissimilarity measure and the cluster module colors in the mRNA expression (minimum number of genes in the module is 30). **(C)** Adjust cut-height as 0.25 (the red line) to cluster the module eigengene. **(D)** Correlation of the co-expressed genes in the turquoise model. Each dot is one gene of the module.

To find the co-expression genes of CDCAs, we applied the WGCNA on the LIHC dataset. From the analysis of the scale independence and mean connectivity, we chose the number 12 as the best soft-threshold power to construct the co-expression module (**Figure 4A**). Through the cut-height and minimal module size, we clustered the genes and cut the tree into nine gene modules (**Figures 4B, C**). Furthermore, the turquoise model contained a total of 2,961 genes and eight genes of CDCAs were all in this model. According to the DEGs analysis, a total of 178 genes were obtained by extracting the turquoise module. After taking 178 genes as the center nodes, we calculated the number of edges connected with CDCAs from the network of the turquoise module. As a result, a total of 126 genes with higher connections with CDCAs were obtained and then considered as the co-expression genes. Notably, the Gleason score (correlation coefficient = 0.41 and P = 2E-120) indicated that there was a reliably positive correlation between the module membership and gene significance in the turquoise module for HCC (**Figure 4D**).

**Figure 5:**
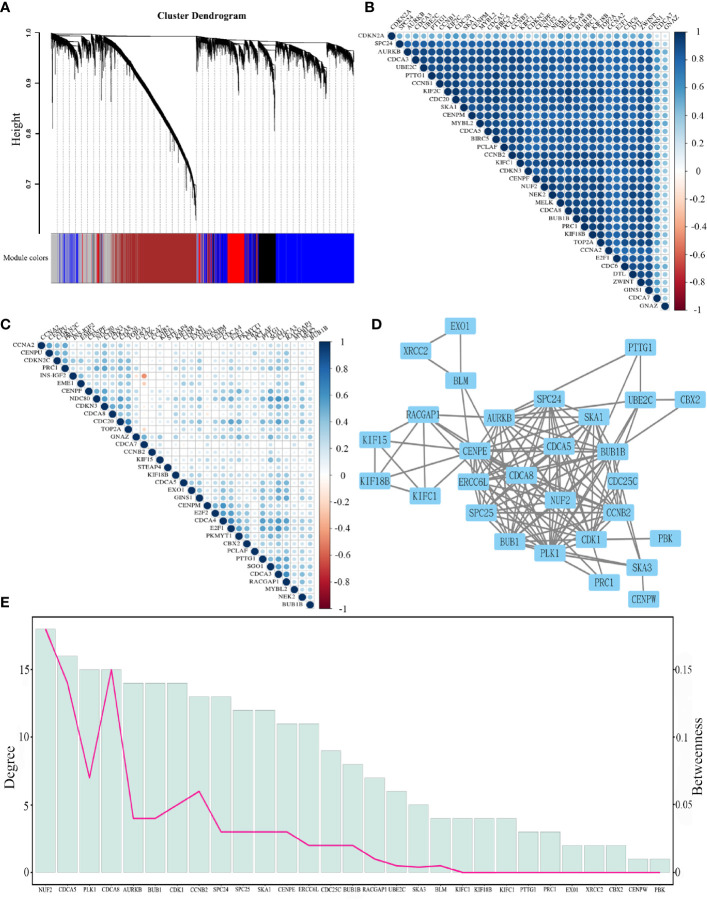
**(A)** Cluster dendrogram of DNA methylation values into 11 modules. **(B)** Heatmap shows the correlation of the top 35 genes from co-expressed and co-methylated genes according to the standard deviation of the mRNA expression. **(C)** Heatmap shows the correlation of the top 35 genes from co-expressed and co-methylated genes according to the standard deviation of the DNA methylation values. **(D)** The protein-protein interaction network for co-expressed and co-methylated genes. **(E)** The degree and betweenness of 29 significant genes. The bar graph shows the degree, and the line graph indicates the betweenness.

In addition, the co-methylation network analysis reveals that the methylation genes of HCC were divided into 11 modules (**Figure 5A**). Particularly, five genes of CDCAs were found in the module colored as “brown” (a total of 1,859 genes). Through the DMP and connection analysis, we got 117 co-methylated genes from the brown module. Furthermore, 71 genes overlapping in the turquoise and brown modules, are regarded as the co-expressed and co-methylated genes. The heatmap showed that there were positive correlations of these genes in the mRNA expression and DNA methylation values (**Figures 5B, C**).

### Function Enrichment Analysis

**Figure 6:**
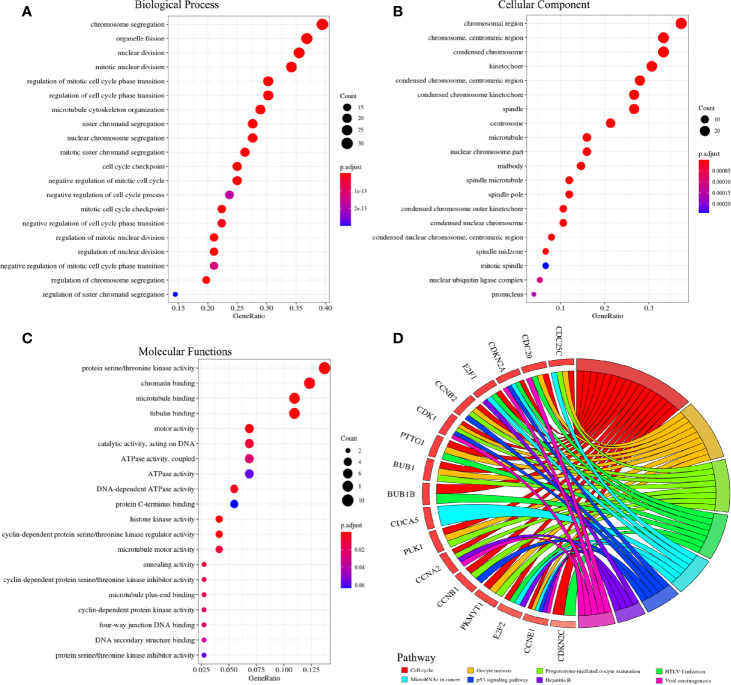
Go and KEGG analysis of 71 co-expressed and co-methylated genes. **(A)** Terms for biological process. **(B)** Terms for cellular component. **(C)** Terms for molecular functions. **(D)** Pathways for KEGG analysis.

After removing the disconnected nodes, the protein interaction network of the co-expressed and co-methylated genes was constructed, consisting of 29 genes and 243 edges (**Figure 5D**). The mean value of the targets per gene is 8.3, which indicated the diversity of these significant genes in biological targeting. Additionally, it is clear that genes with higher degrees would also have higher betweenness (**Figure 5E**), displaying the strong positive correlation between the degree and betweenness (**Supplementary Figure 1**). Notably, NUF2, CDCA5, and CDCA8 had the highest degree and betweenness, which are considered as the center nodes of this network. The GO enrichment analysis reveals that the functions of these target genes are associated with the biological process, cellular component, and molecular functions. Notably, we obtained several significant enrichment terms from the analysis of biological process, such as cell cycle checkpoint and mitotic nuclear division (**Figure 6A**). In addition, the cellular component analysis showed the enrichment of these genes in the chromosome region and condensed chromosome (**Figure 6B**). Also, it is clear that protein serine/threonine kinase activity was the most significant enrichment term in the analysis of molecular functions (**Figure 6C**). As to the KEGG pathway analysis, cell cycle, p53 signaling pathway, HEpatitis B, and viral carcinogenesis had strong associations with these genes (**Figure 6D**). The results of the PPI network and enrichment analysis show the potential association between CDCAs and the immune function. For example, CDCAs have the higher connectivity with the immune-related genes [e.g., CDK1 (36, 37) and PLK1 (38, 39)] in the network, and also significantly enrich in the immune-related terms [e.g., p53 signaling pathway (40–42) and viral carcinogenesis (43–45)].

### Methylation Difference of CDCAs

**Figure 7:**
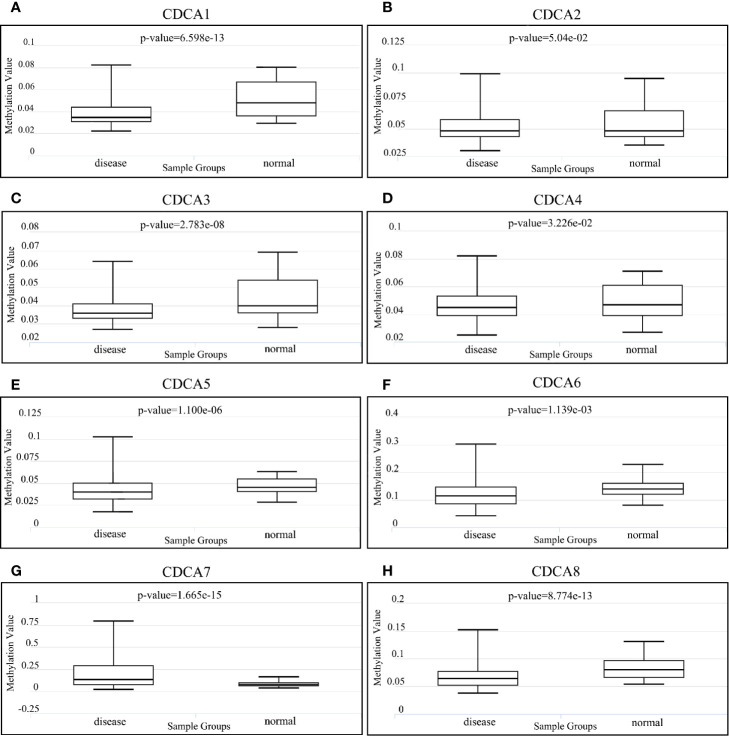
Methylation analysis of CDCAs *via* DiseaseMeth 2.0. Methylation values for **(A)** CDCA1, **(B)** CDCA2, **(C)** CDCA3, **(D)** CDCA4, **(E)** CDCA5, **(F)** CDCA6, **(G)** CDCA7, and **(H)** CDCA8.

In accordance with the result of DiseaseMeth (**Figure 7**), the methylation levels of CDCA1 (P = 6.59E-13), CDCA3 (P = 2.78E-08), CDCA4 (P = 3.22e-02), CDCA5 (P = 1.10E-06), CDCA6 (P = 1.13E-03), and CDCA8 (P = 8.77e-13) are all significantly higher in normal samples than the levels in disease samples, whereas CDCA7 (P = 1.66E-15) was significantly higher in disease samples. Besides, CDCA2 (P = 5.04E-02) had no significant difference in the sample groups. Additionally, further analysis demonstrates that CDCAs have a consistently negative correlation between the expression and methylation levels (**Supplementary Figure 2**).

### Transcriptional Levels of CDCAs Have Strongly Positive Correlations With the Immune Infiltration

**Figure 8:**
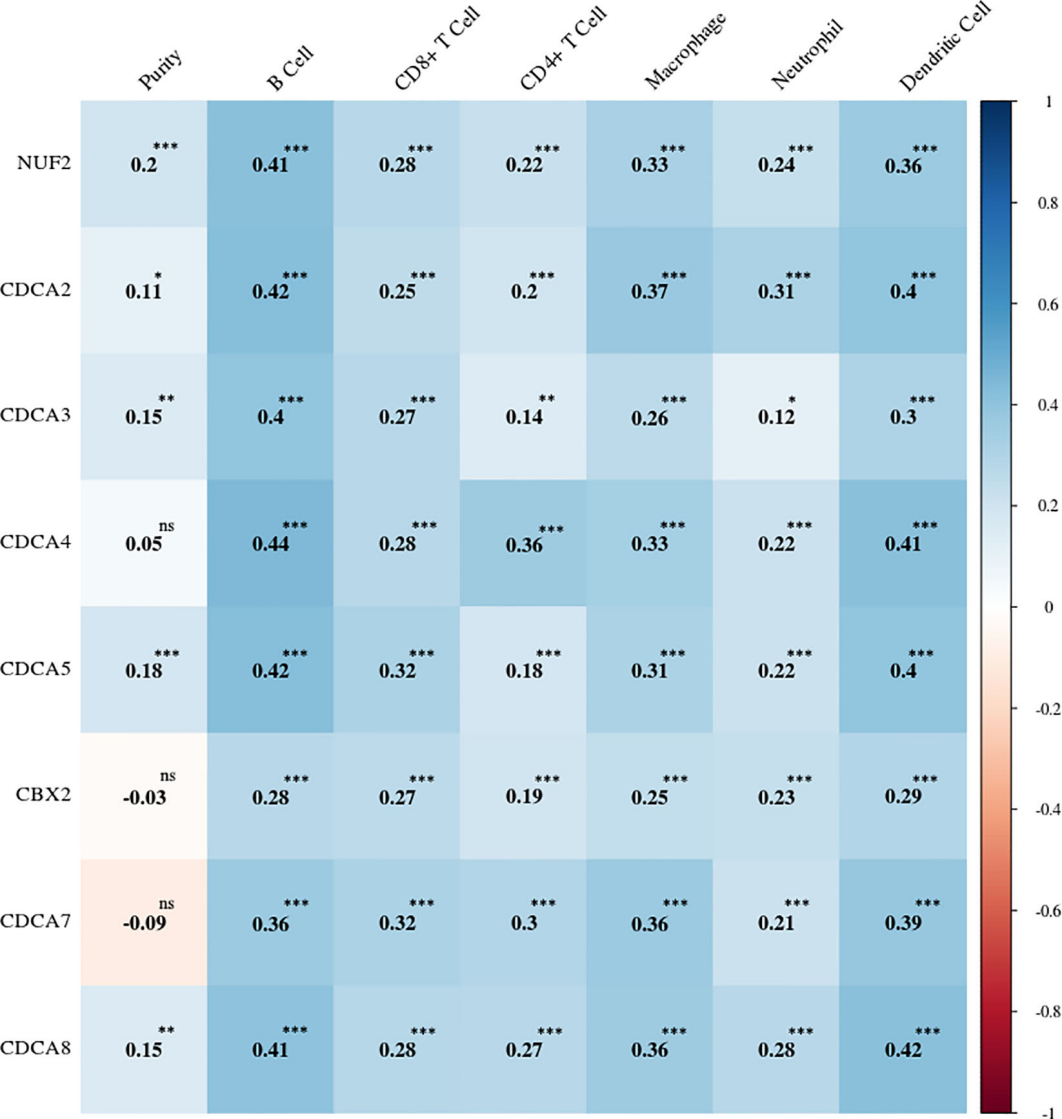
Correlation heatmap between the expression levels of CDCAs and the immune infiltration levels. The number shows the correlation value. ns, no statistical significance; *p < 0.05; **p < 0.01; ***p < 0.001.

The online tool TIMER was applied to investigate potential relations between the mRNA expression levels of CDCAs and the states of the tumor purity and immune cell infiltration. The strongly positive associations (**Supplementary Figure 3**) were observed between CDCA1–8 and six types of immune infiltrates (e.g., B cells and dendritic cells). Meanwhile, CDCA1–5 and CDCA8 demonstrated the weak correlations in the tumor purity, whereas the associations of CDCA6 and CDCA7 were weak and negative (**Figure 8**).

### Association of CDCAs Methylation With Enhancing Tumor Immunogenicity

**Figure 9:**
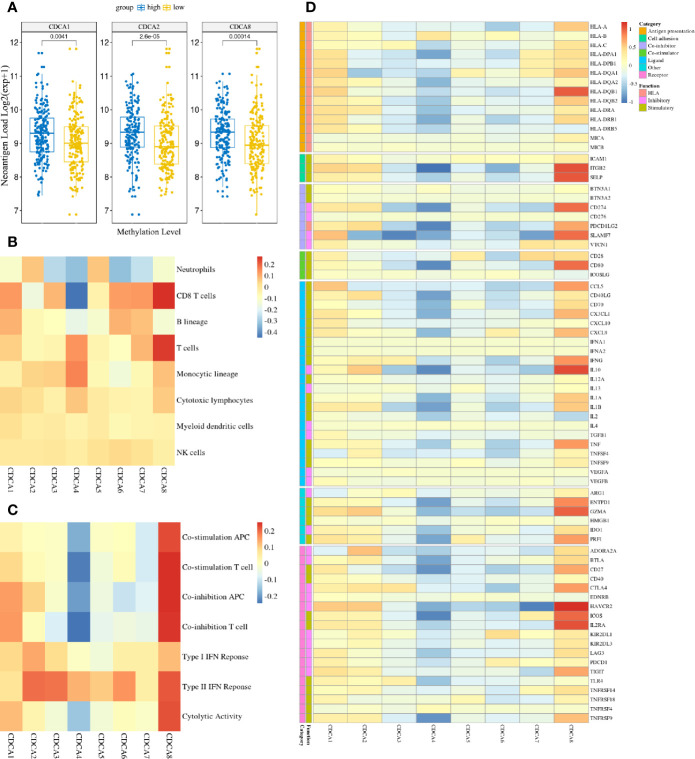
The high-methylation CDCAs have associations with enhancing tumor immunogenicity. **(A)** The difference of neoantigen load between methylation groups of CDCAs in the LIHC cohorts (only show the result with significant differences). **(B)** Heatmap visualizes the log2FC between methylation groups in the MCP-counter scores across CDCAs. **(C)** Heatmap visualizes the log2FC between methylation groups in the ssGSEA scores across CDCAs. **(D)** Heatmap visualizes the log2FC between high and low methylation groups in the immune-related mRNA expressions across CDCAs. The y-axis indicates different immune-related genes and their function and category types. Red suggests up-regulation, while blue suggests down-regulation.

To explore cancer immune infiltrates in HCC, we investigated the tumor immunogenicity between methylation groups of CDCAs. The neoantigen load had significant differences in CDCA1, CDCA2, and CDCA8, which indicated that these genes with low-methylation had powerful associations with weakening tumor immunogenicity (**Figure 9A**). Additionally, several tumor-infiltrating parameters, especially the T cells and cytotoxic lymphocytes, were generally more abundant in the high-methylation samples than those in low-methylation samples across CDCAs (**Figure 9B**). And the results of the immune signature analysis suggest that the type I and type II IFN responses are almost higher in all the CDCAs with high-methylation (**Figure 9C**). Moreover, it also showed that CDCA1, CDCA2, and CDCA8 with high-methylation were more abundant in seven immune signatures. To further estimate the immune profile, we profoundly analyzed the different expression patterns of immune-related genes in methylation states of CDCAs (**Figure 9D**). In consonance with the result of immune infiltrates and gene signatures, different stimulatory immunomodulators had the general up-regulation in CDCA1, CDCA2, and CDCA8 with high-methylation, such as chemokines (CCL5, CX3CL1, CXCL10, CXCL9) and human leucocyte antigen (HLA-A, HLA-DPA1, HLA-DQA1). All these results reveal that the methylation states of CDCA1, CDCA2, and CDCA8 have strong associations in the tumor immunogenicity.

### CDCAs With Low-Methylation Deteriorate the Overall Survival in HCC

The cox regression analyses were conducted to explore the contribution of CDCAs with methylation groups as the independent prognostic signatures of patient survival. Based on the clinical factors, including age, gender, and pathological stage, the result confirms that the predictive ability of CDCAs methylation, including CDCA1–6 and CDCA8, were independent of other clinical characteristics for overall survival in HCC (**Table 1**).

**Table 1:** Cox regression analysis of CDCAs methylation groups and clinical characteristics of patients in the LIHC dataset.

Variables	Univariate analysis		Multivariate analysis	
	HR (95%) CI	P	HR (95%) CI	P
Age (>61/<=61)	1.229 (0.870–1.737)	0.242	1.194 (0.840–1.698)	0.323
Gender (Male/Female)	0.820 (0.575–1.168)	0.271	0.847 (0.591–1.215)	0.367
Stage (I/II)	1.334 (0.817–2.176)	0.25	–	–
Stage (I/IIIA)	2.485 (1.575–3.919)	9.00E-05	–	–
CDCA1	0.598 (0.421–0.850)	4.12E-03	0.605 (0.425–0.861)	5.33E-03
CDCA2	0.687 (0.486–0.972)	3.40E-02	0.678 (0.479–0.958)	2.81E-02
CDCA3	0.665 (0.470–0.941)	2.11E-02	0.672 (0.474–0.951)	2.51E-02
CDCA4	0.682 (0.483–0.964)	3.00E-02	0.680 (0.481–0.963)	3.00E-02
CDCA5	0.706 (0.499–0.998)	4.82E-02	0.721 (0.508–1.023	4.90E-02
CDCA6	0.655 (0.462–0.928)	1.73E-02	0.639 (0.450–0.906)	1.20E-02
CDCA7	1.183 (0.837–1.670)	3.41E-01	1.109 (0.768–1.600)	5.81E-01
CDCA8	0.573 (0.404–0.813)	1.83E-03	0.588 (0.414–0.835)	3.08E-03

HR, hazard ratio; CI, confidence interval; statistical significance P < 0.05.

**Figure 10:**
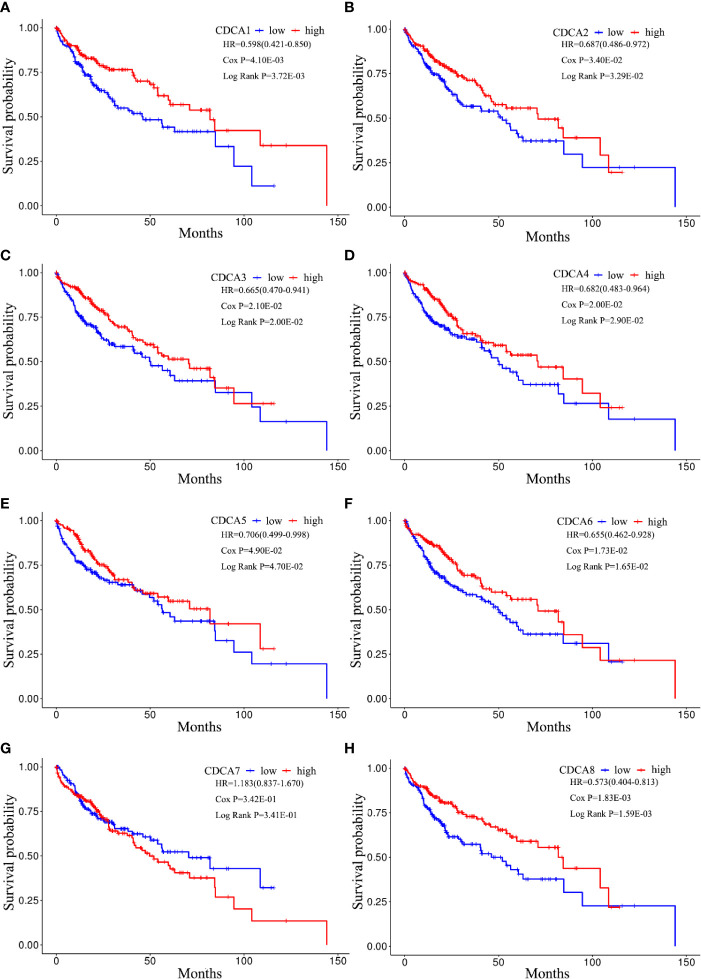
Kaplan-Meier curves for CDCAs methylation groups. Survival analyses for **(A)** CDCA1, **(B)** CDCA2, **(C)** CDCA3, **(D)** CDCA4, **(E)** CDCA5, **(F)** CDCA6, **(G)** CDCA7, and **(H)** CDCA8. Patient samples are dichotomized based on the median methylation levels of each CDCAs. P-value refers to the cox and log-rank tests. HR shows the hazard ratio and 95% confidence interval.

The Kaplan-Meier plotter was applied to visualize the survival of CDCAs methylation groups in the LIHC cohorts. Furthermore, the log-rank and cox tests were applied to evaluate the result. As shown in **Figure 10**, the patients with the high-methylation levels of CDCAs, including CDCA1–6 and CDCA8, extensively had a longer OS than the low-methylation counterparts. Besides, the p-value of the log-rank and cox analysis indicated that the result had significant differences and effectiveness.

## Discussion

The success of immune therapy in cancer treatment has been widely reported (12, 13). As an important strategy for immunotherapy, DNA methylation could be served as a promising target in different cancers, especially in HCC (14, 46, 47). In addition, CDCAs play crucial roles in the development and progression of cancers, such as HCC (7, 10). However, little is known about the relationship between the immune infiltrates and DNA methylation of CDCAs.

Presently, we first explored the mRNA expression data in 19 different types of cancer datasets. As shown in **Figures 2** and **3**, the results indicate that most genes of CDCAs, including CDCA1–3 and CDCA5–8, are significantly up-regulated in diverse malignant cancers, especially in HCC. Indeed, the abnormal expression of CDCAs has been reported in the tumor tissue of HCC (48–50). Additionally, the co-expressed and co-methylated gene networks analysis shows that the biological enrichments of CDCAs are significantly associated with immune functions, such as p53 signaling pathway and viral carcinogenesis (**Figures 4**–**6**). Actually, it is reported that p53 could stimulate the innate immune system to maintain tissue homeostasis and suppress tumorigenesis (40), and CDCA2 also plays a critical role in the activation of p53 (41). Besides, CDCA4 has involvement in regulating the transcriptional activities of p53, and its over-expression could lead to p53-independent growth inhibition (42). All the results demonstrate that CDCAs have a potential association with immune functions.

Moreover, to explore the potential relation of CDCAs and infiltrating immune cells, we analyzed their expression modes and found the significant and positive correlations between the expression levels of CDCAs and proportions of infiltrating immune cells (**Figure 8**). Consistently, it is reported that the knockdown of CDCA5 shows strong inhibition in tumor proliferation, which could be an attractive target for immunotherapy (51). Besides, CBX2 could promote the production of type I interferon in macrophages, which shows the regulation in antiviral immune response (19). Also, the expression of CDCA6 in immune cells (e.g. macrophages and T cells) suggests the intensity of innate immune response (19). In addition, tumor heterogeneity could be a significant obstacle for the tumor diagnosis and immunotherapy treatment in HCC, especially the methylations variability (46, 52–54).

Additionally, our study indicated that CDCAs (i.e., CDCA1, CDCA3–6, and CDCA8) have significant hypomethylation levels in tumor tissues of HCC (**Figure 7**). Notably, the consistently negative correlation between the expression and methylation levels of CDCAs (Supplementary **Figure 2**) shows that the abnormal methylation can cause the expression complexity of CDCAs, which play important roles in regulating the biological functions of CDCAs. In fact, the methylation of CDCAs has been partly reported in HCC, but the association with infiltrating immune would still be unclear (20, 55, 56). For instance, Cai et al (55). have demonstrated that CDCA5, as a differentially methylated gene, can be served as a new novel therapeutic target for HCC. In another study, it is also revealed that the abnormal methylation and over-expression of CDCA8 plays an essential role in the regulation of cell cycle (56, 57), while the dysregulated cell cycle is a significant hallmark of immune checkpoint pathways for immunotherapy (58). With respect to CDCA7, it has been reported that CDCA7 can be involved in the regulation of T cells, through dynamic DNA methylation (20, 21). Thus, we explored the link between CDCAs methylation and immune states in HCC and found that the methylation levels of CDCAs have strong associations in tumor immunogenicity (**Figure 9**). For example, the neoantigen load, as the mutated protein from the somatic mutation of tumors, participates in the function of immune responses, which would be considered as a novel marker for immunity therapy (59–61). Herein, it is indicated that CDCAs, especially CDCA1, CDCA2, and CDCA8, have higher levels of neoantigen load in the high methylation group (**Figure 9A**). In addition, the immune cell infiltration and significant markers of immune cells (**Figures 9B–D**) suggest the improvement of immune activity under the high methylation status of CDCAs, which is consistent with the obvious result of neoantigen load. Besides, the survival curve analysis also shows that CDCA1–6 and CDCA8 with low-methylation levels significantly deteriorate the overall survival of patients in HCC (**Figure 10**), further suggesting that methylation levels of CDCAs can be regarded as the predictive biomarkers for determining prognosis and immune infiltration in HCC. All the results provide valuable evidence to the combination of the DNA methylation of CDCAs and immune infiltration in HCC.

However, our study also has some limitations. Due to the data type requirements, including mRNA expression, methylation expression, and neoantigen load calculation, we only obtained the data from TCGA, which may cause the data bias of this investigation. Therefore, more tumor samples and further experimental validation are necessary to perform for evaluating the biological roles of CDCAs in HCC.

## Conclusion

Collectively, we identified 71 co-expressed and co-methylated genes of CDCAs that were enriched in biological terms related to immune function. And we integratively analyzed the association between the immune infiltrates and methylation of CDCAs. Our results demonstrate that the methylation groups of CDCAs are valuable in predicting prognosis. Thus, the methylation of CDCAs could be novel predictive biomarkers for immune infiltrates and be used in the clinical diagnosis and treatment of HCC.

## Data Availability Statement

The datasets presented in this study can be found in online repositories. The names of the repository/repositories and accession number(s) can be found in the article/**Supplementary Material**.

## Author Contributions

YW conducted the bioinformatics analysis, prepared the figures, and wrote the paper. YW, assisted by HG, TO, LZ, JH, and SH, contributed to the experiments of the manuscript. YY and HK conceived the idea, designed the study, and coordinated the project. All authors contributed to the article and approved the submitted version.

## Conflict of Interest

The authors declare that the research was conducted in the absence of any commercial or financial relationships that could be construed as a potential conflict of interest.
