# Supplementary material for: Comprehensive Analysis of CDCAs Methylation and Immune Infiltrates in Hepatocellular Carcinoma
Source: Front Oncol. 2021 Feb 16;10:566183. doi: 10.3389/fonc.2020.566183 (PMC7921702; doi:10.3389/fonc.2020.566183)
Supplement: Supplementary file 1 [file DataSheet_1.docx]

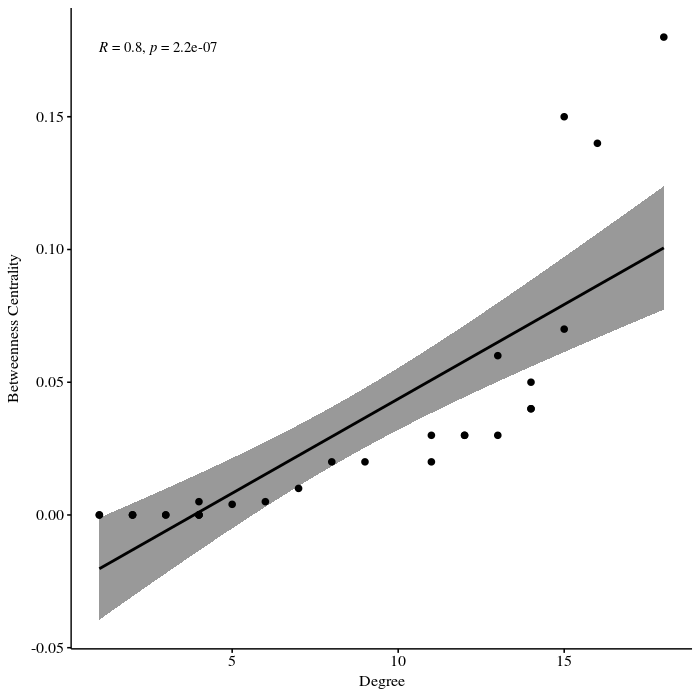


**Supplementary Figure 1.** Correlation of the betweenness centrality and degree among 29 significant genes. The R value shows the pearson correlation coefficient. The p value suggests the statistical significance.


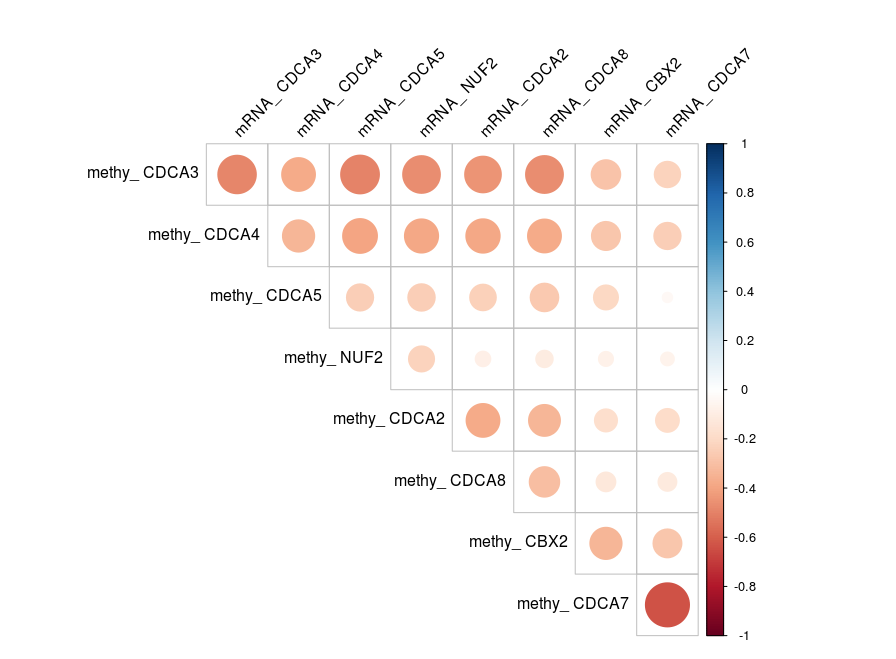


**Supplementary Figure 2**. Correlation between expression and methylation levels of CDCAs. Red and blue colors show the negative and positive correlation. The area of point indicates the correlation value.


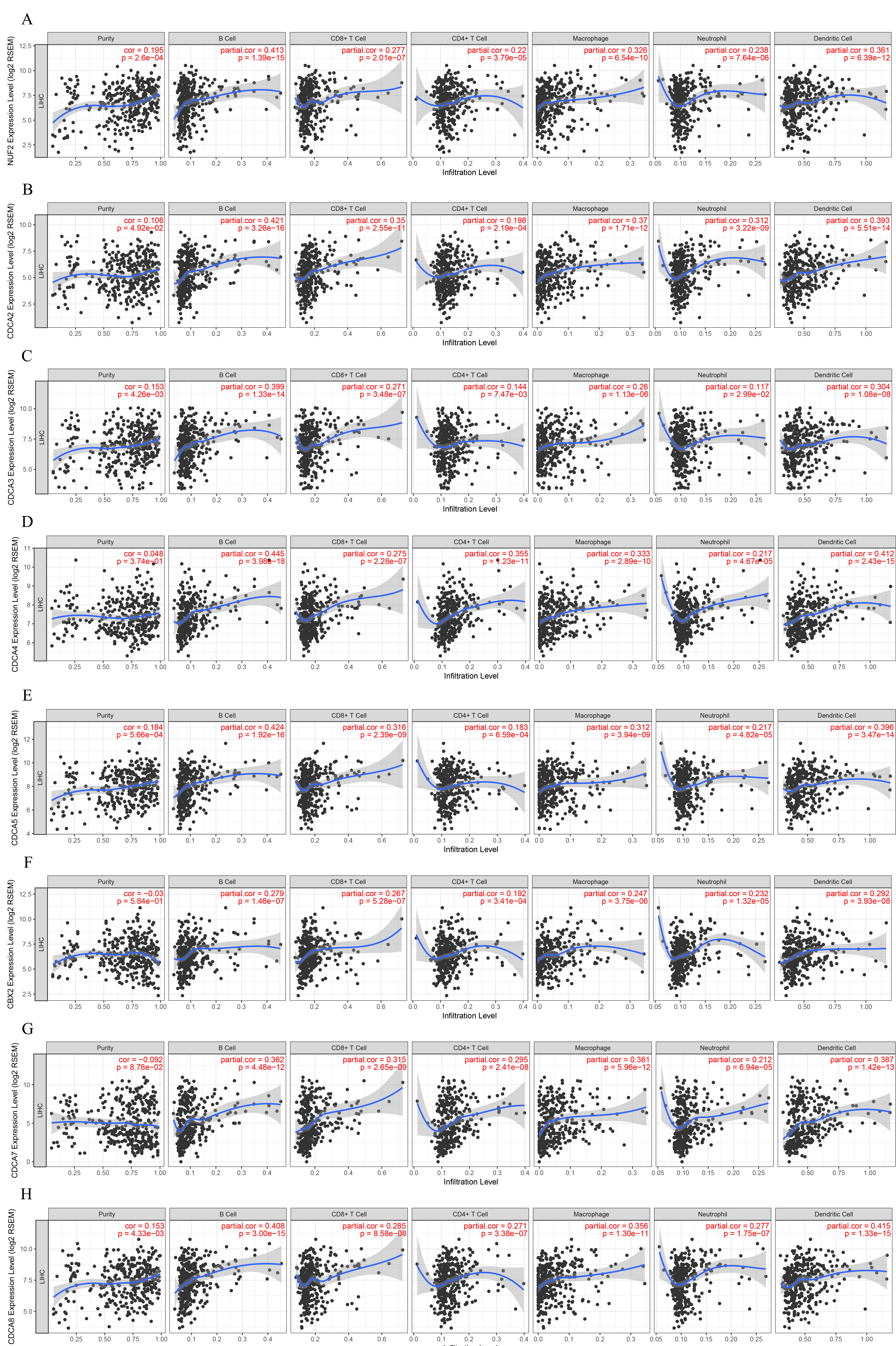


**Supplementary Figure 3.** Correlation between the expression levels of CDCAs and the immune infiltration levels. Correlation analysis for **(A)** CDCA1, **(B)** CDCA2, **(C)** CDCA3, **(D)** CDCA4, **(E)** CDCA5, **(F)** CDCA6, **(G)** CDCA7, and **(H)** CDCA8. The points show the expression value of HCC samples.
